# Supplementary material for: OsOFP6 Overexpression Alters Plant Architecture, Grain Shape, and Seed Fertility
Source: Int J Mol Sci. 2024 Mar 1;25(5):2889. doi: 10.3390/ijms25052889 (PMC10931697; doi:10.3390/ijms25052889)
Supplement: Supplementary file 1 [file ijms-25-02889-s001.zip › ijms-2879295-supplementary.pdf]

Supplementary Material

OsOFP6 Overexpression Alters Plant Architecture, Grain Shape, and Seed Fertility

Xuting Zhu [1,2,†], Yuan Li [1,2,†], Xiangqian Zhao [1], Yukai Feng [1,2], Zhengkai Bao [1], Wenzhen Liu [2],* and Feifei Li [1],*

[1]College of Advanced Agricultural Science, Zhejiang A&F University, Hangzhou 311300, China; zxt1477702045@163.com (X.Z.); lylylyyqq@163.com (Y.L.); zhaoxq@zafu.edu.cn (X.Z.); fengyukai0103@163.com (Y.F.); 15088793603@163.com (Z.B.)

[2] State Key Laboratory of Rice Biology and Breeding, China National Rice Research Institute, Hangzhou 311400, China

***** Correspondence: liuwenzhen@caas.cn (W.L.); lifei-fei@163.com (F.L.)

† These authors contributed equally to this work.

Supplementary Tables

Supplementary Table S1. Primers used in TAIL PCR.

Primer	Primer sequence（5′-3′）
TL1	TCCCAGATAAGGGAATTAGGGTTCCTA
TL2	GTTCCTATAGGGTTTCGCTCATGTGTTG
TL3	CCAGTACTAAAATCCAGATCCCCCGA
AD1	NTCGA(G/C)T(A/T)T(G/C)G(A/T)GTT
AD2	NGTCGA(G/C)(A/T)GANA(A/T)GAA
AD3	(A/T)GTGNAG(A/T) ANCANAGA
AD4	NGTA(G/C)A(G/C)(A/T)GTNA(A/T)CAA

Supplementary Table S2. Primers were used to knock out vector.

Primer	Primer sequence（5′-3′）
gRNA1	GTTGGCGCGCCGCCCGGGGAGGTG
gRNA2	AAACCACCTCCCCGGGCGGCGCGC
gRNA3	GTTGGACCGAGAAGAGGCCCGACA
gRNA4	AAACTGTCGGGCCTCTTCTCGGTC

SupplementaryTable S3. Primers were used to construct overexpression vectors.

Primer	Primer sequence（5′-3′）
OsOFP6-Flag-*Bam*H I	GGATCCATGGGGAGGCACAAGTTCAG
OsOFP6-Flag-*Kpn* I	GGTACCCATCTTGATGTCCGCGAGGT

Supplementary Table S4. Primers used in this study for gene expression analysis by qRT-PCR.

**Primer**	**Primer sequence（5′-3′）**
ACTIN1-F	TGCTATGTACGTCGCCATCCAG
ACTIN1-R	AATGAGTAACCACGCTCCGTCA
RT-OsOFP6-F	CGTCGACGTGTTCGAGCATA
RT-OsOFP6-R	GGATTCACCATGCAAGACAAGA
Q-OsCPS-F	TCTCCCCTTCAGCTACTGCT
Q-OsCPS-R	ACGCCTCCGTCGAATTTCTT
Q-OsKAO-F	CTTCCTCCATCATTTCTCC
Q-OsKAO-R	AAGCAGTTGTCCACAGGC
Q-GA3ox1-F	TGATTGCAATTATCCATGCCAG
Q-GA3ox1-R	TCCTGGTTCTACAGTGAAACTC
Q-OsGA13ox-F	AGAAGTGGAGAAAGACTACGG
Q-OsGA13ox-R	CAATGATCTTTCTCTGGTGTGC
Q-GA20ox3-F	CTACCGGTCGGACACCAACA
Q-GA20ox3-R	TTTGCTTGATCCAGGCGACG
Q-OsGID2-F	AGAGAGCCGTGGTAATGAGG
Q-OsGID2-R	AAGACCTTGGACTCTGGAGC
Q-SLR1-F	GACGTCAACGAACGCTCAATT
Q-SLR1-R	CGGAGTCCAGTCGTCGATCT
Q-D2-F	GCTCAAGAGGCGAAAAACCG
Q-D2-R	ACCGCAGCGTCTCTGTTATC
Q-D11-F	TCACTGCTCCAGGTATGGGA
Q-D11-R	ATGAATTGGCCTGGGGTAGC
Q-CPD-F	CCCACCACACGAACTCTCAA
Q-CPD-R	AAGAGCAGCTGCAAAACGAC
Q-DWARF-F	CACCAGGGAGAAGCTCACAG
Q-DWARF-R	TCTTCGGGCGCTTTACCTTT
Q-DWARF4-F	GAAGAGCGGGTTGAGAAGC
Q-DWARF4-R	CAGAGCCCATCCGAGAAGA
Q-BZR1-F	CCATGGAGATAGAAGGGACGG
Q-BZR1-R	GTCACCCTCCCCTTGTCGAA

Supplementary Table S5. Primers were used for hygromycin verification.

**Primer**	**Primer sequence（5′-3′）**
HPT-F	TGTTGGCGACCTCGTATTGG
HPT-R	CTTCGATGTAGGAGGGCGTG

Supplementary Table S6. Primers were used for coseparation analysis.

**Primer**	**Primer sequence（5′-3′）**
P1	AAGATCGCAATTCGCAAGGT
P2	CGTAGGATCGTGTGGTAATGAA
P3	CGTCCGAGGGCAAAGAAATAG

Supplementary Table S7. Primers were used to construct subcellular localization vector.

**Primer**	**Primer sequence（5′-3′）**
OsOFP6-GFP-*Bam*H I	TCTAGACATGGGGAGGCACAAGTTCAG
OsOFP6-GFP-*Xba* I	GGTACCTTGAGACTGAGCTCTTCCG

Supplementary Table S8. Primers were used to construct protein interaction vectors.

Y-OsOFP6-F	atatggccatggaggccGAATTCATGGGGAGGCACAAGTTCAGG
Y-OsOFP6-R	atgctagttatgcggccgCTGCAGCTACATCTTGATGTCCGCGAGG
Y-E3BB-F	CCGGAATTCTCATCAATGGCAACTCCA
Y-E3BB-R	CGCGGATCCCACAACGCAAACATCACC
BI-OsOFP6-F	tggcgcgccactagtggatccATGGGGAGGCACAAGTTCAGG
BI-OsOFP6-R	agcggtaccctcgaggtcgacCATCTTGATGTCCGCGAGGT
BI-E3BB-F	tggcgcgccactagtggatccATGGCAACTCCAAATACCTATTCA
BI-E3BB-R	agcggtaccctcgaggtcgacCCCAAATACCTCCTCATTGCA
cLU-OsOFP6-F	tacgcgtcccggggcggtaccATGGGGAGGCACAAGTTCAGG
cLU-OsOFP6-R	acgaaagctctgcaggtcgacCTACATCTTGATGTCCGCGAGG
nLU-E3BB-F	cgagctcggtacccgggatccATGGCAACTCCAAATACCTATTCA
nLU-E3BB-R	cgcgtacgagatctggtcgacCCCAAATACCTCCTCATTGCA

Supplementary Figures

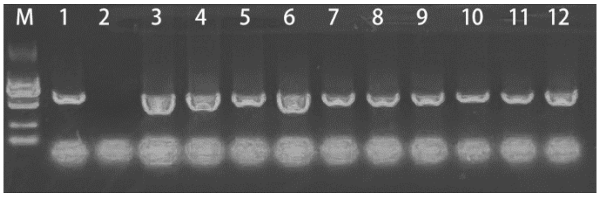

Supplementary Figure S1. PCR assay revealed that the mutant was co-isolated with T-DNA insertion. M: marker 2000bp, 1: positive control, 2: wild-type plant, 3-12: mutants
